# Small-molecule compounds inhibiting S-phase kinase-associated protein 2: A review

**DOI:** 10.3389/fphar.2023.1122008

**Published:** 2023-04-05

**Authors:** Jia Jing, Li Rui, Sun Junyuan, Yang Jinfeng, Hong Zhihao, Lu Weiguo, Jia Zhenyu

**Affiliations:** ^1^ Schools of Laboratory Medicine and Bioengineering, Hangzhou Medical College, Hangzhou, Zhejiang Province, China; ^2^ Women’s Hospital, School of Medicine, Zhejiang University, Hangzhou, Zhejiang Province, China; ^3^ School of Pharmacy, Hangzhou Medical College, Hangzhou, Zhejiang Province, China; ^4^ Key Laboratory of Women′s Reproductive Health Research of Zhejiang Province, Hangzhou, Zhejiang Province, China; ^5^ Institute of Occupation Diseases, Hangzhou Medical College, Hangzhou, Zhejiang Province, China

**Keywords:** cancer treatment, S-phase kinase-associated protein 2, inhibitor, E3 ubiquitin ligase, SCF^Skp2^

## Abstract

S-phase kinase-associated protein 2 (Skp2) is a substrate-specific adaptor in Skp1-CUL1-ROC1-F-box E3 ubiquitin ligases and widely regarded as an oncogene. Therefore, Skp2 has remained as an active anticancer research topic since its discovery. Accordingly, the structure of Skp2 has been solved and numerous Skp2 inhibiting compounds have been identified. In this review, we would describe the structural features of Skp2, introduce the ubiquitination function of SCF^Skp2^, and summarize the diverse natural and synthetic Skp2 inhibiting compounds reported to date. The IC_50_ data of the Skp2 inhibitors or inhibiting compounds in various kinds of tumors at cellular levels implied that the cancer type, stage and pathological mechanisms should be taken into consideration when selecting Skp2-inhibiting compound for cancer treatment.

## 1 Introduction

S-phase kinase-associated protein 2 (Skp2), a member of the F-box family of proteins, was discovered by Zhang et al. (1995). Skp2 functions as a substrate-specific adaptor in Skp1-CUL1-ROC1-F-box (Lyapina et al., 1998) E3 ubiquitin ligases, and many proteins playing antioncogenic roles are recognized by Skp2 and tagged with ubiquitin by SCF^Skp2^(Lyapina et al., 1998; Tsvetkov et al., 1999). For example, Skp2 mediates the K48-linked ubiquitination of p27 and p21, both of which are tumor suppressors, resulting in the proteasomal degradation of these proteins and cell-cycle promotion (Yu et al., 1998; Tsvetkov et al., 1999). In addition, overexpression of Skp2 is observed in numerous human cancers (Gstaiger et al., 2001; Shigemasa et al., 2003). Accordingly, Skp2 is regarded as an oncogene and has remained a highly active topic in cancer-therapy research since its discovery.

Protein p27, cyclin dependent kinase inhibitor 1B, was previously found to bind and inhibit cyclin-CDK to arrest the cell cycle, and recently p27 was also reported to function as an oncogene to involve in tumor migration, invasion and regulation of gene expression in tumor (Razavipour et al., 2020). Skp2 was a rate-limiting factor of the degradation of p27 protein (Carrano et al., 1999; Tsvetkov et al., 1999), and the Skp2-p27 axis controlled S phase entry, tumor stemness as well (Wang J. et al., 2021). The upstream regulation factors of Skp2-p27 axis have also been reported, such as nuclear casein kinase and cyclin dependent kinase substrate 1(NUCKS1) (Hume et al., 2021), focal adhesion kinase 1 (Jeong et al., 2022), DnaJ heat shock protein family member C5(Li et al., 2021; Wang H. et al., 2021). Various Skp2 inhibiting compounds have been developed to inhibit the degradation of p27 mediated by Skp2 for cancer treatment, such as Skp2E3LIs (Wu L. et al., 2012), and the p27 protein level was taken as a point for selecting Skp2 inhibiting compound, such as when screening SMIP004 (Rico-Bautista et al., 2010). We referred to these compounds in this manuscript.

Since been reported to regulate cell cycle, promoting cell proliferation *via* p27 (Carrano et al., 1999), Skp2 was discovered to have various other functions in tumor pathological processes, including apoptosis (Nakayama et al., 2000), DNA repair (Wu J. et al., 2012), tumor metastasis (Li et al., 2004), and senescence (Lin et al., 2010; Wang et al., 2012).

Regarding the apoptosis regulation, Skp2 regulates various signaling pathways *via* binding and ubiquitination of its substrates to affect cellular apoptosis. For example, programmed cell death protein 4 is ubiquitinated and degraded by SCF^Skp2^, and its degradation inhibits translation of p53 directly, leading to cell apoptosis (Li et al., 2019). In another case, the activation of TNF-related apoptosis-inducing ligand receptor 1/2(TRAIL-R1/R2 (DR4/DR5) death receptors induce forming of the death-inducing signaling complex (DISC) and lead to the cell apoptosis. Overexpression of Cullin-1/Skp2 enhances the ubiquitination of CASP8 and FADD like apoptosis regulator (FLIP), a DISC member, and this ubiquitination promotes turnover of FLIP between long form and short form, resulting modulation of TRAIL-R2-mediated apoptosis (Roberts et al., 2020). In Rb1-deficient tumors, Skp2 regulates apoptosis by limiting E2F transcription factor 1(E2F1) activity *via* a pRb-Skp2-p27-cyclin A-E2F1 pathway(Lu et al., 2014).

Skp2 affects cellular DNA repair in both homologous recombination (HR) and Non-homologous end joining (NHEJ) manners. In response to DNA double-strand breaks, Skp2 promotes K63-linked ubiquitination of Nijmegen breakage syndrome protein 1(NBS1), a vital event for facilitating ataxia telangiectasia mutated (ATM) protein recruitment to the DNA foci and activation (Wu J. et al., 2012). The knocked down of Skp2 also decreases the protein level of Ku70, which contributes to the NHEJ pathway (Jia et al., 2020).

As far as tumor metastasis and senescence is concerned, dysregulation of Skp2 level reduces osteosarcoma cell invasion and lung metastasis both *in vitro* and *vivo* (Ding et al., 2017; Zhang et al., 2018). Knockdown of Skp2 also attenuates the migration of colon carcinoma and gastric cancer cells (Wei et al., 2013; Chen et al., 2014). Skp2 orchestrates with Myc to recruit p300 to the promoter of zinc finger E-box binding homeobox 1 (Zeb1), which leads to induction of Zeb1 transcription, finally promoting tumor migration and invasion(Wang et al., 2022). Skp2 also mediates the ubiquitination and degradation of Amino-terminal enhancer of split (AES), which is a tumor and metastasis suppressor, and enhances tumor metastasis (Wang Z. et al., 2021). Downregulation of Skp2 also induces senescence in glioma (Wu et al., 2020). The ERK/SKP2/p27 pathway played important roles in cellular senescence induced by 14-3-3β depletion in glioblastoma cells (Seo et al., 2018). In addition to degradation of p21 and p27, Skp2 also regulates cell senescence by cascade. Skp2 decreased the K63-linked ubiquitination of histone modification enzyme Jumonji/ARID Domain-Containing Protein 1B (JARID1B) by E3 ubiquitin ligase TRAF6, and inactivation of Skp2 induces senescence through JARID1B accumulation in cells (Lu et al., 2015).

The AMPK-SKP2-CARM1 pathway is involved in the autophagy regulation after nutrient starvation, and targeting Skp2 leads to activation of autophagy (Shin et al., 2016). Skp2 mediated the ubiquitination and degradation of autophagy regulator Beclin1 (BECN1) to decrease autophagy, and this inhibitory effect of Skp2 on autophagy was regulated by a phosphorylation cascade involving HSP90 cochaperone FK506 binding protein (FKBP)51, PH domain leucine-rich repeat protein phosphatase (PHLPP) and AKT1 (Gassen et al., 2019).

Emerging evidence showed that Skp2 highly correlated with drug resistance and poor prognosis, rendering Skp2 as a therapeutic target (Wu et al., 2021). Skp2 contributes to drug resistance by disturbing various cellular procedures, such as cell proliferation and apoptosis, cell cycle, transcription, et al. Skp2 has been reported to involve in the resistance of various drugs, such as paclitaxel, cisplatin, tamoxifen, and mTOR inhibitor. Family with sequence similarity 60A (FAM60A) mediates the cisplatin-resistant in human lung adenocarcinoma A549 cells, and it exerts its function *via* upregulating expression of Skp2 and the following inhibition of cell death as well (Hou et al., 2020). POU Domain Transcription Factor OCT4 mediates the tamoxifen resistance in breast cancer cells, and its expression is repressed by NK3 Homeobox 1(Nkx3-1), which is a substrate protein of Skp2 (Bhatt et al., 2016).

In addition, Skp2 is also involves in metabolism regulation (Chan et al., 2012). Activation of Epidermal Growth Factor (EGF) signaling pathway induces AKT localize to mitochondrion to interact with Hexokinases (HK)2, and promote HK2 mitochondrial localization consequently. As a substrate protein of Skp2, the K63-linked ubiquitination of AKT mediated by SCF^Skp2^ is required in this signaling pathway (Yu et al., 2019). The decreased Skp2 protein level reprogrammes cellular aerobic glycolysis. Protein Isocitrate Dehydrogenase (NADP(+)) (IDH) 1/2, a substrate protein of Skp2, promotes the cell metabolic fluctuation between glycolysis and tricarboxylic acid (TCA) cycle, by its Skp2-dependent protein fluctuation in cell cycle. Skp2 inhibition leads to IDH1/2 accumulation and promotes cell metabolism shift from glycolysis to TCA cycle. This Skp2–IDH1 signaling axis is also involved in the cancer “Warburg” metabolic phenotype (Liu J. et al., 2021).

The serine/threonine kinase AKT, one upstream regulator of Skp2, increases the expression of Skp2 mRNA, and it also modified by Skp2 post-translationally. The phosphoinositide-3-kinase (PI3K)/AKT signaling pathway promotes E2F1 bind to the promoter of SKP2 gene (Reichert et al., 2007), relying on maintaining the c-myc expression in a glycogen synthase kinase-3 (GSK3)-dependent fashion (Schild et al., 2009), thus increases the expression levels both of Skp2 mRNA and protein. AKT also binds and phosphorylates Skp2, triggering SCF^Skp2^ E3 ligase formation. This phosphorylation of Skp2 also promotes Skp2 localize to the cytosol (Lin et al., 2009), preventing ubiquitination and degradation of Skp2 (Gao et al., 2009). Meanwhile, the Skp2 mediates the K63-linked ubiquitination of AKT (Gao et al.), which is required for epidermal growth factor receptor (ErbB receptor)-mediated AKT activation (Chan et al., 2012). This ubiquitination of AKT associates closely with functions of AKT protein in tumor development, such as cell membrane recruitment (Wang G. et al., 2019), mitochondrial localization (Yu et al., 2019), especially increasing the Skp2 level by this AKT-Skp2 positive feedback loop.

In addition to AKT-Skp2, there are other feedback loops reported involve Skp2. The Skp2 autoinduction loop, i.e., a self-amplifying feedback loop, increases Skp2 mRNA and protein level in G1 phase, which contains Skp2, p27, retinoblastoma 1 (RB) and E2F (Yung et al., 2007). During the process of human papillomavirus (HPV) infection, Skp2 was involved in a negative feedback loop of E2-Skp2. The HPV-18 E2 protein inhibits function of anaphase-promoting complex/cyclosome (APC/C) ubiquitin ligase, leading to Skp2 accumulation. E2 itself is a substrate of Skp2 and ubiquitinated by the increased Skp2 protein finally, resulting in the expression of E6 and E7(Bellanger et al., 2010).

Skp2 itself is also degraded by ubiquitin-proteasome system. In cell cycle, ubiquitin ligase anaphase-promoting complex/cyclosome and its activator Cdh1(Cdh1/APC) has been reported to ubiquitinate Skp2 and promote its degradation in G1 phase (Bashir et al., 2004). This degradation is regulated by the phosphorylation of Skp2 *via* cyclin dependent kinase 2 (CDK2) and cell division cycle 14B (Cdc14B) (Rodier et al., 2008). Upon TGFβ stimulation, Skp2 translocates from cellular cytosol to nucleus, and then is ubiquitinated by Cdh1/APC, which is critical for TGFβ cytostatic effect (Hu et al., 2011). The tumor suppressor F-Box and WD-40 domain-containing protein 2 (FBXW2) was also proved to ubiquitinate Skp2 and promote its degradation. The axis β-TrCP-FBXW2-SKP2 are related to cancer cell growth regulation (Xu et al., 2017).

The stability of Skp2 protein is also regulated by deubiquitylases (DUBs). USP10(Liao et al., 2019), USP13(Chen et al., 2011), USP2(Zhang et al., 2021), and USP14(Wu et al., 2022) was reported to deubiquitylate Skp2. The binding between USP2 and Skp2 disrupts the Skp2-substrate binding, and stabilizes both Skp2 and substrates (Zhang et al., 2021). Various transcription factors have been reported to regulate Skp2 expression transcriptionally, such as selenocysteine tRNA gene transcription-activating factor, Forkhead box protein O3 (FOXO3a) (Wu et al., 2013; Shin et al., 2016), NUCKS1(Hume et al., 2021).

Skp2 relates to several important cellular signaling pathways, such as Hippo, WEE1(Pan et al., 2021), autophagy and so on. The Hippo pathway effector yes-associated protein (YAP) induces the acetylation of Skp2 to suppress cell ploidy and tumorigenesis (Zhang et al., 2017), while YAP is also ubiquitinated by Skp2 to induce its nuclear localization and transcriptional activity (Yao et al., 2018). Skp2 also promotes the ubiquitination and mitochondrial localization of AKT, to regulate cell glycolysis and proliferation (Yu et al., 2019). In autophagy pathway, Skp2 regulates the level of co-activator-associated arginine methyltransferase 1 in the AMPK-SKP2-CARM1 signaling cascade to regulate cell autophagy (Chen et al., 2008; Shin et al., 2016).

The structure of Skp2 has been described. It has an F-box domain, a typical structural feature of F-Box family proteins. This 40-amino-acid-residue F-box motif is located next to the N-terminal region, which comprises ∼100 amino acids. Three non-canonical leucine-rich repeats (LRRs) act as “linkers” connecting the F-box to seven other canonical LRRs, which act as protein-protein interaction modules, directly binding to substrates. Finally, the C-terminal tail is located next to the LRR structure (Schulman et al., 2000; Zheng et al., 2002).

The SCF^Skp2^ complex is a RING E3 ubiquitin ligase characterized by the existence of a zinc-binding RING domain. In this complex, a cullin protein acts as a scaffold, binding the RING-box protein Rbx at its N terminus and the adaptor Skp1 and substrate receptor Skp2 at its C terminus (Zheng et al., 2002; Chan et al., 2013).

Before a protein is ubiquitinated, the ubiquitin molecule is activated upon forming a highly reactive thioester bond between its C terminus and the active site Cys of the ubiquitin-activating enzyme (E1). This process, which forms an E1–Ub complex, is catalyzed by E1 and is ATP-dependent. Then, the activated ubiquitin is transferred onto the conserved catalytic Cys residue of ubiquitin-conjugating enzyme (E2) by a transthiolation reaction, generating an E2-Ub complex. The E2 interacts with SCF^Skp2^ E3 ligase, in which Skp2 recognizes and binds with the substrate protein. Finally, the substrate protein is positioned into the gap between Skp2 and E2, indicating that the SCF^Skp2^ E3 ligase acts as scaffold to orient the E2–ubiquitin complex close to the substrate protein, facilitating the transfer of the ubiquitin from the E2 to the substrate directly and efficiently. The Skp2 protein determines the substrate protein and the specificity of this ubiquitination (Scheffner et al., 1995; Morreale and Walden, 2016; Asmamaw et al., 2020).

Until recently, Skp2 has shown the ability to conjugate both K48-linked and K63-linked ubiquitin chains on its substrates, such as C/EBPα (Thacker et al., 2020) and NBS1 (Wu J. et al., 2012). The K48-linked ubiquitination promotes the degradation of substrates, while the K63-linked ubiquitination enhances the stability and modulates the function of the substrate (Deng et al., 2000; Cai et al., 2020).

Skp2 was reported to be overexpressed in various aggressive cancers both at mRNA level and protein level since its discovery, and the protein level of Skp2 was reported to associate with the prognosis in various cancer patients. Due to its close relation with cancers, Skp2 was regarded as an attractive target for cancer therapy. However, besides cancers, Skp2 was also reported to associate with other non-cancer diseases, such as chronic kidney disease (Suzuki et al., 2011), pulmonary Fibrosis(Li et al., 2016; Mikamo et al., 2018), systemic dysregulation of COVID-19 patients(Gassen et al., 2021), and asthma(Park et al., 2016; Liu B. et al., 2021) as well. In our manuscript, we would like to focus on the Skp2-inhibiting compounds that was used to treat tumors experimentally.

In the last 20 years, numerous attempts have been made to develop antitumor drugs targeting Skp2 proteins, and various Skp2 inhibitory compounds have been developed. However, none has been proved suitable for clinical use. In this review, we introduce the structural features of Skp2, discuss the ubiquitination functionality of SCF^Skp2^, and summarize the diverse range of natural and synthetic Skp2 inhibitors or inhibitory compounds reported to date.

## 2 Synthetic small-molecule Skp2-inhibiting compounds

The synthetic small-molecule Skp2-inhibiting compounds were separated into two groups, i.e., “structure-based” and “non-structure-based”. In the “structure-based” group, all the reported compounds were selected by structure-based approached, in which the compound binds with Skp2 and disrupts the protein interaction, thus inhibiting Skp2’s functions directly. In the ‘non-structure-based’ group, the compounds were identified by various methods, leading to different mechanisms when related to how compounds worked.

As an inhibitor should be able to bind to the target protein directly, in this manuscript, the Skp2-inhibiting compound which binds Skp2 directly and disrupts the binding between Skp2 and other proteins is called Skp2 inhibitor, while the compound only inhibits the expression or activity of Skp2 is called Skp2 inhibitory compound.

### 2.1 Structure-based compounds

#### 2.1.1 SZL-P1-41

Compound SZL-P1-41, also known as #25, has been reported as a small structure-based inhibitor of Skp2. Nineteen residues within the Skp2 protein have been reported to mediate the binding between Skp2 and Skp1, and these residues constitute two distinct pocket-like regions. Chemical compounds that target these two pocket-like regions have been identified using structure-based high-throughput virtual screening, and compound SZL-P1-41 was selected from 120,000 compounds (Table 1).

**TABLE 1:** Structures of some of the Skp2 inhibitors or inhibiting compounds in this review.

Name	Structure	Name	Structure
SZL-P1-41 Cas No. 222716-34-9	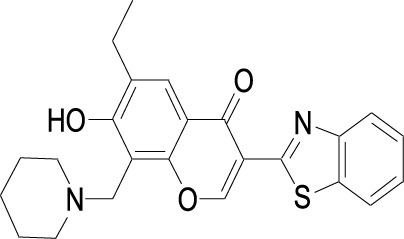	C2(Skp2E3LI)	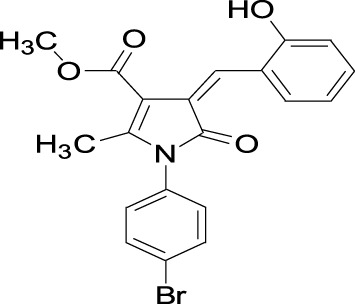
C20(Skp2E3LI)	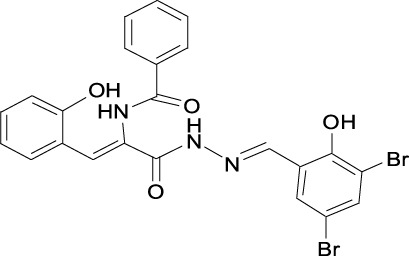	SKPin C1 Cas No. 432001- 69-9	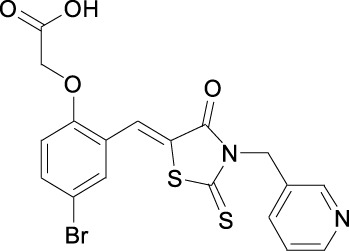
SMIP004 Cas No. 143360-00-3	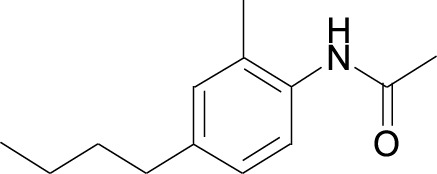	ABT-751 Cas No. 857447- 92-8	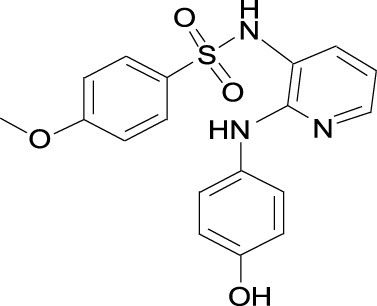
Rottlerin Cas No. 82-08-6	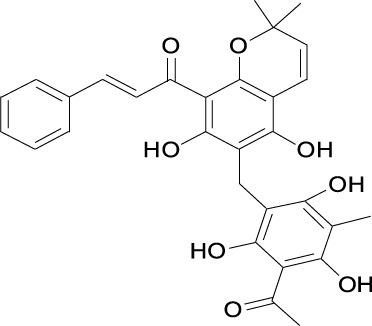	Longikaurin A Cas No. 75207- 67-9	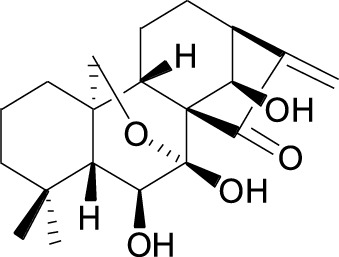
Curcumin Cas No .458-37-7	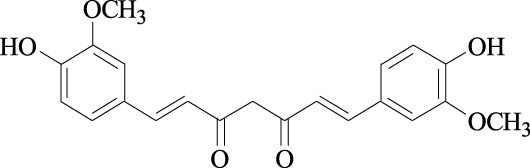	Flavokawain A Cas No.3420-72- 2	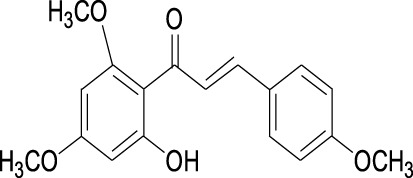
Gartanin Cas No. 33390-42-0	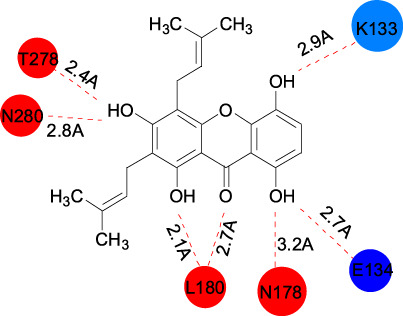	Betulinic acid Cas No.472-15-1	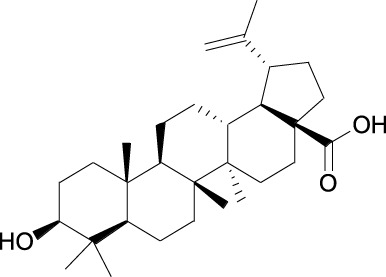
5gg Cas No. 14937-32-7	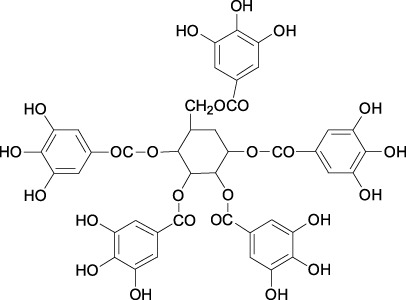	Quercetin Cas No. 117-39-5	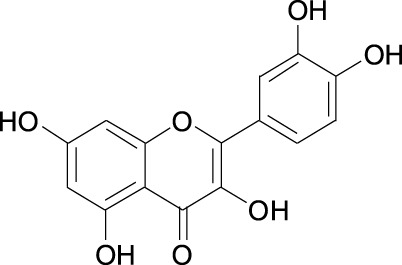
Lycopene Cas No. 502-65-8	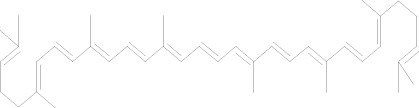	Safranal Cas No. 116-26-7	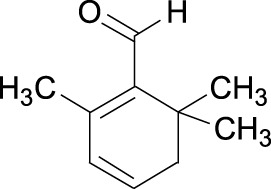
Linichorin A	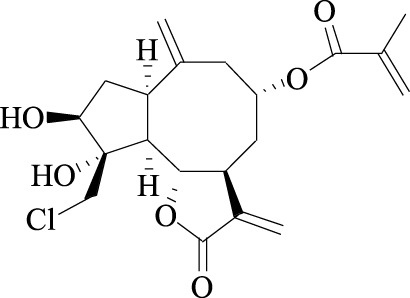	Gentian violet Cas NO.548-62-9	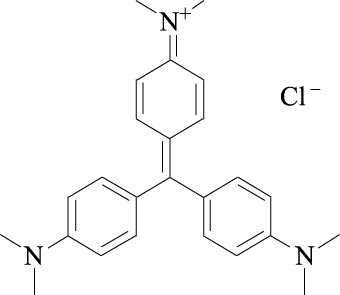

Compound SZL-P1-41 binds specifically to the Trp97 and Asp98 residues of Skp2, preventing Skp2–Skp1 interaction and thus inhibiting Skp2 activity. Treatment with SZL-P1-41 was found to trigger p53-independent cellular-senescence and inhibit cellular aerobic glycolysis *in vitro*, leading to inhibition of prostate cancer stem cell populations and self-renewal. SZL-P1-41 was also reported to inhibit prostate- and lung-tumor growth dose-dependently *in vivo*(Chan et al., 2013), as well as TAIL7 cells *in vitro* and *in vivo*(Rodriguez et al., 2020) (Table 2).

**TABLE 2:** Inhibitory effects of the Skp2 inhibitors or inhibiting compounds in various kinds of tumors at cellular level.

**Tumor type**	**Cell line**	**Compound**	**Treatment time**	IC_50_
Prostate Carcinoma				
	PC3	SZL-P1-41	4 days	5.61 μM(Chan et al., 2013)
	Parental PC3	Gartanin	72 h	13.56 ± 0.20 μM(Pham et al., 2020)
	quiescent PC-3	Safranal	72 h	0.109 ± 0.002 mM(Jiang et al., 2020)
	proliferative PC-3	Safranal	48 h	0.512 ± 0.038 mM(Jiang et al., 2020)
	LNCaP	SZL-P1-41	4 days	1.22 μM(Chan et al., 2013)
	LNCaP-S14	SMIP004	72 h	1.09 μM(Rico-Bautista et al., 2010)
	LNCaP-WT	SMIP004	72 h	40 μM(Rico-Bautista et al., 2010)
	quiescent LNCaP	Safranal	72 h	0.133 ± 0.034 mM(Jiang et al., 2020)
	proliferative LNCaP	Safranal	48 h	0.234 ± 0.023 mM(Jiang et al., 2020)
	parental 22Rv1	Gartanin	72 h	8.32 ± 0.18 μM(Pham et al., 2020)
Lung Carcinoma				
	H460	SZL-P1-41	4 days	5.15 μM(Chan et al., 2013)
		SKPin C1	72 h	33 ± 12 μM(Zhao et al., 2020)
		MLN4924	72 h	0.43 ± 0.08 μM(Zhao et al., 2020)
		Flavokawain A	72 h	19 ± 8.9 μM(Zhao et al., 2020)
	A549	SZL-P1-41	4 days	5.36 μM(Chan et al., 2013)
		SMIP004	24 h	500 nM(Huang et al., 2017)
	H1299	SZL-P1-41	4 days	10.5 μM(Chan et al., 2013)
	H3255	SZL-P1-41	4 days	5.38 μM(Chan et al., 2013)
	H520	SKPin C1	72 h	7.3 ± 2.1 μM(Zhao et al., 2020)
		MLN4924	72 h	>10 μM(Zhao et al., 2020)
		Flavokawain A	72 h	18 ± 11 μM(Zhao et al., 2020)
	H69	SKPin C1	72 h	1.2 ± 0.6 μM(Zhao et al., 2020)
		MLN4924	72 h	8.7 ± 1.1 μM(Zhao et al., 2020)
		Flavokawain A	72 h	9.9 ± 2.3 μM(Zhao et al., 2020)
	H146	SKPin C1	72 h	5.3 ± 0.8 μM(Zhao et al., 2020)
		MLN4924	72 h	0.15 ± 0.02 μM(Zhao et al., 2020)
	H196	SKPin C1	72 h	8.0 ± 2 μM(Zhao et al., 2020)
		MLN4924	72 h	12 ± 8 μM(Zhao et al., 2020)
	H720	SKPin C1	72 h	0.85 ± 0.15 μM(Zhao et al., 2020)
		MLN4924	72 h	0.015 ± 0.005 μM(Zhao et al., 2020)
	Primary mouse SCLC lung cell	SKPin C1	72 h	0.70 ± 0.05 μM(Zhao et al., 2020)
		MLN4924	72 h	1.8 ± 0.21 μM(Zhao et al., 2020)
		Flavokawain A	72 h	8.3 ± 4.1 μM(Zhao et al., 2020)
	Primary mouse SCLC liver metastatic cell	SKPin C1	72 h	0.63 ± 0.16 μM(Zhao et al., 2020)
		MLN4924	72 h	0.18 ± 0.08 μM (Zhao et al., 2020)
		Flavokawain A	72 h	12 ± 2.7 μM(Zhao et al., 2020)
Hepatocellular Carcinoma				
	HepG2	Longikaurin A	36 h	5.13 μM(Liao et al., 2014)
	Hep3B	SZL-P1-41	4 days	9.84 μM (Chan et al., 2013)
	SMMC-7721	Longikaurin A	36 h	2.75 μM(Liao et al., 2014)
	BEL-7402	Longikaurin A	36 h	6.83 μM(Liao et al., 2014)
	Huh7	Longikaurin A	36 h	7.12 μM(Liao et al., 2014)
Breast Carcinoma				
	MCF-7/HER2	Flavokawain A	72 h	13.6 μM(Jandial et al., 2017)
	MCF10A	Flavokawain A	72 h	>100 μM (Jandial et al., 2017)
	SKBR3	Flavokawain A	72 h	10 μM (Jandial et al., 2017)
	tsFT210	Linichlorin A	48 h	1.6 μM(Ooi et al., 2013)
		Gentian violet	48 h	0.6 μM(Ooi et al., 2013)
Osteosarcoma				
	U2OS	SZL-P1-41	4 days	5.41 μM(Chan et al., 2013)
	SaOS-2	Flavokawain A	72 h	7.5 μg/mL(Zhang et al., 2018)
	Saos-LM7	Flavokawain A	72 h	7.5 μg/mL(Zhang et al., 2018)
	143B	Flavokawain A	72 h	7.5 μg/mL (Zhang et al., 2018)
Cervical cancer				
	HeLa	Linichlorin A	48 h	3.2 μM(Ooi et al., 2013)
		Gentian violet	48 h	0.4 μM(Ooi et al., 2013)
Leukemia T cell leukemia				
	TAIL7	SZL-P1-41	96 h	30 μM(Rodriguez et al., 2020)
		SKPin C1	96 h	2.4 μM(Rodriguez et al., 2020)
	Molt4	SKPin C1	96 h	2.6 μM(Rodriguez et al., 2020)
	HPB-ALL	SKPin C1	96 h	2.0 μM(Rodriguez et al., 2020)
	SupT1	SKPin C1	96 h	1.9 μM(Rodriguez et al., 2020)
	JurKat	SKPin C1	96 h	1.6 μM(Rodriguez et al., 2020)
	CEM	SKPin C1	96 h	1.1 μM(Rodriguez et al., 2020)
	Loucy	SKPin C1	96 h	1.1 μM(Rodriguez et al., 2020)
	Human primary T-ALL	SKPin C1	48 h	1.72 μM(Rodriguez et al., 2020)
Myeloid Leukemia				
	HL-60	Linichlorin A	72 h	1.2 ± 0.6 μM(Estevez-Sarmiento et al., 2020)
	U-937	Linichlorin A	72 h	1.9 ± 0.5 μM(Estevez-Sarmiento et al., 2020)
	U-937/Bcl-2	Linichlorin A	72 h	2.9 ± 1.8 μM(Estevez-Sarmiento et al., 2020)
	K562	Diosmetin	48 h	8.42 μM(Liu et al., 2020a)
	KBM5	Diosmetin	48 h	8.52 μM(Liu et al., 2020a)
	KBM5-T3151	Diosmetin	48 h	6.46 μM(Liu et al., 2020a)
Urinary bladder Carcinoma				
	TCCSUP	Flavokawain A	48 h	10.55 μM (Tang et al., 2008)
	HT1197	Flavokawain A	48 h	7.9 μM (Tang et al., 2008)
	5637	Flavokawain A	48 h	13.1 μM (Tang et al., 2008)
	T24	Flavokawain A	48 h	16.7 μM (Tang et al., 2008)
	UMUC3	Flavokawain A	48 h	17.7 μM (Tang et al., 2008)
	HT1376	Flavokawain A	48 h	14.7 μM (Tang et al., 2008)
	J82	ABT-751	24 h	>3 μM(Dehghanian et al., 2021)
		ABT-751	48 h	0.7 μM(Dehghanian et al., 2021)
		ABT-751	72 h	0.37 μM (Dehghanian et al., 2021)
	BFTC905	ABT-751	24 h	>3 μM (Dehghanian et al., 2021)
		ABT-751	48 h	0.6 μM (Dehghanian et al., 2021)
		ABT-751	72 h	0.4 μM (Dehghanian et al., 2021)
Glioblastoma				
	U251	Curcumin	72 h	15 μM (Wang et al., 2015)
	SNB19	Curcumin	72 h	15 μM (Wang et al., 2015)
Melanoma				
	SK-MEL-1	Linichlorin A	72 h	3.6 ± 1.3 μM
	MUM2B	SKPin C1	4 days	0.86 μM (Zhao et al., 2019)
	OM431	SKPin C1	4 days	1.83 μM (Zhao et al., 2019)
Endometrial Carcinoma				
	ECC-1	Skp2E3Li C2	48 h	EC50 = 14.3 μM (Pavlides et al., 2013)
Colorectal Carcinoma				
	HCT116	7-azaindoles (Compound 5)	72 h	EC50 = 4.7 μM (Altmann et al., 2017)
		7-azaindoles (Compound 6)	72 h	EC50 = 4.9 μM (Altmann et al., 2017)
Pancreatic Carcinoma				
	Patu8988	Curcumin	72 h	10 μM (Su et al., 2016b)
	Panc-1	Curcumin	72 h	15 μM (Su et al., 2016b)

^a^LNCaP-S14: positive LNCaP clone with stable Skp2 overexpression U-937/Bcl-2: U-937 cell overexpressing Bcl-2.

#### 2.1.2 Skp2E3LIs

Skp2E3LIs are small-molecule inhibitors that target SCF-Skp2/Cks1 E3 ligase. Several Skp2E3LIs have been identified by molecular docking based on the crystal structure of Skp2/Cks1 and virtual library screening (Wu L. et al., 2012) (Table 1). Skp2E3LI C2 and C20 have been demonstrated to block proliferation of endometrial carcinoma cells by inhibiting nuclear p27 ubiquitinational degradation. In E2-primed mice, treatment with Skp2E3LI C2 was found to inhibit tumor cellular proliferation by stabilizing nuclear p27 and decreasing the proliferation of endometrial epithelial cells(Pavlides et al., 2013) (Table 2).

#### 2.1.3 SKPin C1

SKPin C1 is an Skp2 inhibitor identified by *in silico* virtual ligand screening against the Skp2-p27 binding pocket in the Skp2 protein (Table 1). SKPin C1 specifically targets the Skp2-p27 interaction interface to block their binding (Wu L. et al., 2012), resulting in the decreased ubiquitination of p27 and cellular G1 phase arrest (Zhao et al., 2019).

SKPin C1 has also been reported to inhibit uveal melanoma and lung cancer growth both *in vitro* and *in vivo* (Zhao et al., 2020) (Zhao et al., 2019) (Table 2); trigger cell apoptosis in metastatic melanoma and breast cancer *in vitro*; and inhibit proliferation of murine primary T-ALL cells with wildtype Skp2, as well as several human T-ALL cell lines (Rodriguez et al., 2020) (Table 2). In addition, SKPin C1 has also been reported to inhibit cell proliferation in U266 and RPMI 8226 myeloma cells and to trigger apoptosis by increasing cleaved caspase-3 protein levels (Yang et al., 2019). However, SKPin C1 does not inhibit the proliferation of SKP2 knock-out embryonic fibroblasts (Rodriguez et al., 2020). In a related study, the inhibitory effects of SKPin C1 were found to be dose-dependent when treating pRb and p53 doubly deficient primary prostate tumor cells with p27T187A KI mutation (Zhao et al., 2017).

#### 2.1.4 DT204

DT204 is an Skp2 inhibitor identified by virtual and computational screening against multiple myeloma RPMI8226 cells. DT204 prevents Skp2 from incorporating into the SCF^Skp2^ complex by reducing Skp2 binding to Cullin-1, and treatment with DT204 increases p27 protein levels. In proteasome inhibitor BTZ resistant multiple myeloma patients, the SCF^Skp2^ components were expressed at greater levels, and correlated with poor prognosis. And in BTZ-resistant cells, the number and activity of proteasome increased both. The knockdown of Skp2 expression sensitized BTZ-resistant multiple myeloma cells to proteasome inhibitors. In a study with multiple myeloma xenografts with MM1.S-luciferase-expressing cells, combined treatment of DT204 (10 mg/kg) and the proteasome inhibitor BTZ inhibited tumor growth (Malek et al., 2017).

### 2.2 Non-structure-based compounds

In this group, the compounds were identified by various methods. Some of the compounds were selected by inhibitory-effect-based methods. However, it is not known for sure whether such compounds bind to Skp2 directly. For example, some of these compounds have been reported to inhibit Skp2 mRNA expression by mechanisms on transcriptional level. We subclassed these compounds by the inhibiting mechanisms into the group of “transcriptional inhibition”, “post-translational inhibition” and “other”.

#### 2.2.1 Transcriptional inhibition

##### 2.2.1.1 Menin inhibitors

Menin is an epigenetic calcium-sensing regulator of Skp2 in colorectal cancer (CRC) that binds the SKP2 promoter. The overexpression of menin upregulates transcription of SKP2 by increasing active histone marks (H3K4me3), and this ability is calcium sensitive and impaired when the cytosolic calcium increased. In CRC tumors, combined menin inhibition and iEGFR treatment induces CRC cell apoptosis *in vitro* and inhibits CRC tumor xenograft growth *in vivo* (Katona et al., 2019), as iEGFR treatment induces calcium release to repress promoting SKP2 transcription by menin.

##### 2.2.1.2 MLN4924

MLN4924 (Pevonedistat) is an SCF^Skp2^ complex neddylation inhibitor, which blocks CUL neddylation and inactivates SCF^Skp2^ complex. MLN4924 also downregulates Skp2 expression transcriptionally *via* regulation of YAP (Lan et al., 2022). Treatment with MLN4924 has been shown to decrease Skp2 protein levels in prostate cancer cell lines PC3, PC3, DR12, and LAPC4 as well as decreasing Slug expression, indicating that neddylation blockade is a potential therapeutic approach for advanced prostate cancer (Mickova et al., 2021). Furthermore, MLN4924 has been reported to cause cell death in small-cell lung cancer organoids, and a significant increase in Skp2 protein levels upon MLN4924 treatment has also been reported, resulting from the inhibited assembling of SCF^Skp2^ complex by MLN4924, in the case the Skp2 protein is not part of SCF^Skp2^ E3 ligase (Zhao et al., 2020).

##### 2.2.1.3 Vemurafenib

Vemurafenib (PLX4032) is a small inhibitor of BRAF V600E, which selectively binds to the ATP-binding site of BRAF V600E kinase and inhibits the activity of this mutated serine-threonine kinase BRAF (Garbe and Eigentler, 2018). Recently, Vemurafenib was reported to inhibit growth of BRAF^V600E^ human melanoma cells, by the mechanisms that inhibition of BRAF^V600E^ suppresses the expression level of c-Myc transcription factor, which binds to the E-box region on SKP2 promoter and regulates the Skp2 expression transcriptionally, dictating cell survival finally. Thus, the Vemurafenib downregulates Skp2 expression transcriptionally *via* regulation of c-Myc (Wu et al., 2020). As Skp2 was reported to correlate with poor outcome in BRAF^V600E^ other than BRAF^WT^ melanomas, targeting Skp2 might be promising in the treatment of BRAF^V600E^ melanomas.

Vemurafenib has been used in clinical trials for treatment with BRAF-mutant melanoma, lung cancer, refractory or relapsed Hairy-Cell Leukemia, and other cancers as well, single or combined with other anti-cancer drugs (Mazieres et al., 2020; Tiacci et al., 2021; Schmitt et al., 2022). However, in BRAF-mutant melanoma, the prolonged use of vemurafenib or other BRAF inhibitors would lead to drug resistance. Skp2 was found to over-express in vemurafenib-resistant melanoma cells, and the stability of Skp2 was related to the drug-resistant mechanisms. Targeting Skp2, combined use of Skp2 inhibitor and Skp2 degradation promoting drugs, was proved to be a solution to BRAF inhibitor resistance melanoma (Wu et al., 2022).

#### 2.2.2 Post-translational inhibition

##### 2.2.2.1 Azaindoles

Azaindoles is considered to be potential Skp2 inhibitory compound post-translationally. The activities of cullin-RING E3 ubiquitin ligases, including SCF^Skp2^, are regulated by the zinc metalloprotease CSN5 (a subunit of COP9 signalosome), relying on the deneddylation function of CSN5, and inhibition of CSN5 promotes the ubiquitination and degradation of Skp2, leading to decreased Skp2 protein levels. Azaindoles have been identified as CSN5 inhibitors using time-resolved fluorescence energy transfer (TR-FRET)-based protease activity assays. Accordingly, treatment of azaindole in human colon carcinoma cell line HCT116 has been found to decrease Skp2 protein levels in a dose-dependent manner and inhibit cell proliferation in CellTiter-Glo assays, along with the phenomenon of deneddylation inhibition demonstrated by trapping of Cul1 in the neddylated status, supporting that azaindoles are Skp2 inhibitory compound, which induced autoubiquitination and degradation of Skp2 (Altmann et al., 2017) (Table 2).

##### 2.2.2.2 ABT-751

ABT-751, an anti-microtubule drug (Table 1), has been reported to inhibit *SKP2* transcription by suppression of the AKT serine/threonine kinase–nuclear factor kappa B signaling pathway and to downregulate stable/phospho-SKP2 post-translationally by inhibition of AKT signaling in the urinary bladder urothelial carcinoma cell lines BFTC905 and J82 (Dehghanian et al., 2021) (Table 2).

##### 2.2.2.3 b-AP15

b-AP15 is a small inhibitor of deubiquitinating enzymes USP14/UCHL5, and treatment with b-AP15 induces tumor regression and upregulates p27 protein level in tumors with p53 deficiency (Jiang et al., 2022). In addition, the treatment with b-AP15 enhances anti-tumor effect in vemurafenib-resistant melanoma (Wu et al., 2022). The mechanisms involved that USP14 stabilized Skp2 by preventing its ubiquitination and degradation, and the inhibition of USP14 by b-AP15 regulated Skp2 expression level post-translationally. The combination treatment with vemurafenib and b-AP15 inhibits cell viability and tumor progression in vemurafenib-sensitive and vemurafenib-resistant melanoma (Wu et al., 2022).

#### 2.2.3 Other

##### 2.2.3.1 SMIP004

The Skp2 inhibitory compound SMIP004 was identified from 7368 compounds by a high-throughput cell-based assay using an LNCaP-derived cell line overexpressing Skp2, taking endogenous nuclear p27 protein level as an endpoint. So, in fact SMIP004 prolonged the half-life of p27 protein. SMIP004 was reported to induce cancer-cell-selective apoptosis by downregulating Skp2 (Table 1). Recently, SMIP004 was reported to disrupt mitochondrial respiration by inducing mitochondrial ROS formation, involving its second roles in inducing cell apoptosis (Rico-Bautista et al., 2013).

SMIP004 has been demonstrated to inhibit the cell cycle in LNCaP, PC3, and DU145 cells (Rico-Bautista et al., 2010) (Table 2). Furthermore, treatment with SMIP004 sensitizes non-small-cell-lung-cancer cell lines A549 and NCI-H1975 to Paclitaxel (Huang et al., 2017) (Table 2). In the field of mental illness, young mice treated with SMIP004 exhibit antidepressant-like activities *via* stabilization of Xic1 by inhibition of Skp2, enhancing neurogenesis in the hippocampus (Wang D. et al., 2019).

##### 2.2.3.2 Imatinib and GNF-5

Imatinib (Gleevec, Glivec) and GNF-5 are both tyrosine kinase inhibitors (TKI), and are primarily designed to target the breakpoint cluster region-Abelson murine leukemia (Bcr-Abl) fusion gene, which is constitutively activated in chronic myeloid leukemia (CML). Both drugs have been proved to treat CML or other solid tumors clinically, however their anti-tumor mechanisms remained unclear. Bcr-Abl recruits p300 to the Sp1 site of SKP2 promoter, up-regulating Skp2 mRNA expression, and Imatinib inhibits this recruitment, leading to decreased Skp2 mRNA. Recently, treatment with Imatinib and GNF-5 has been reported to suppress the expression of Skp2 (Chen et al., 2009), and induce G0/G1 cell cycle arrest, increase p27 and p21 protein levels, inhibiting cell growth in hepatocellular carcinoma (HCC) (Zhang et al., 2020), implying that these two drugs could be used as a potential Skp2-inhibiting compounds indirectly. However, further work is needed to explain the mechanisms of these two TKIs.

### 2.3 Natural Skp2-inhibiting compounds

In this group, we also subclassed these natural compounds by the inhibiting mechanisms into the group of “transcriptional inhibition”, “post-translational inhibition” and “disruption protein interaction”.

#### 2.3.1 Transcriptional inhibition

##### 2.3.1.1 Diosgenin

Diosgenin, a steroidal sapogenin, is extracted from fenugreek seed. In the breast cancer cell lines MCF-7 and MDA-MB-231, diosgenin downregulates the expression of Skp2 at both the mRNA and protein levels, thus decreasing breast cancer cell viability and invasion, as well as stimulating apoptosis (Liu et al., 2020b).

##### 2.3.1.2 Rottlerin

Rottlerin (Kamala, Mallotoxin), a natural polyphenolic compound, is derived from *Mallotus philipinensis* (Table 1). Rottlerin was originally regarded as protein kinase C δ inhibitor (Hong et al., 2019). However, in breast cancer cell lines MCF-7 and MDA-MB-231, pancreatic cancer cell lines Patu8988 and Panc1, rottlerin treatment inhibits tumor cell proliferation, migration and invasion, which is due to downregulated Skp2 expression at mRNA and protein levels (Su et al., 2016a; Yin et al., 2016).

##### 2.3.1.3 Longikaurin A

Longikaurin A (LK-A), an *ent-*Kaurane-type diterpenoid, was extracted from *Isodon ternifolius* (Table 1)*.* LK-A was demonstrated to decrease Skp2 mRNA expression, thereby activating ROS/JNK/c-Jun signaling (Liao et al., 2014). In human HCC cell lines SMMC-7721 and HepG2, LK-A treatment has been shown to induce cell cycle arrest and apoptosis, and it has also been shown to inhibit tumor growth in an SMMC-7721 xenograft model *in vivo*(Table 2).

##### 2.3.1.4 Curcumin

Curcumin has been extracted from *Curcuma longa* (Table 1). Treatment with curcumin inhibited Skp2 mRNA expression, increased p21 and p27 protein levels, inhibited cell proliferation, and induced cell apoptosis in several tumors, such as human head and neck squamous cell carcinoma cell lines(Khan et al., 2018), lung cancer cell line H460 (Chiang et al., 2015), breast cancer cell line MDA-MB-231 (Jia et al., 2014), human glioma cell line U251 and SNB19 (Wang et al., 2015) (Table 2), human nasopharyngeal carcinoma cell lines CNE1 and CNE2 (Feng et al., 2017), and pancreatic cancer cell lines Patu8988 and Panc-1 (Su et al., 2016b) (Table 2). Furthermore, curcumin treatment has been shown to block cell migration in MDA-MB-231 breast cancer cells with Her2/Skp2 overexpression (Sun et al., 2012).

In MDA-MB-231 cells, curcumin treatment inhibited the phosphorylation of AKT, thus decreased the activation of AKT, and finally downregulated the expression of Skp2 at both protein and mRNA levels *via* the PI3K/AKT-Skp2 signaling cascades. However, in MCF-7 cells, the treatment of curcumin resulted in activation of AKT by substantial increase in AKT phosphorylation, and this activation of AKT was not agreement with the upregulated Skp2 expression induced by curcumin treatment, indicating another unknown curcumin-targeted signaling pathway involved in Skp2 regulation in curcumin-resistant MCF-7 cells, rather than PI3K/AKT-Skp2 signaling cascades (Jia et al., 2014).

However, curcumin can modulate multiple cellular signaling pathways, and regulate many downstream targets besides Skp2 to exert its multiple functions like antioxidant, anti-inflammatory and anticancer. For example, in addition to suppressing Skp2 to induce apoptosis, curcumin also activates redox reactions within cells to upregulate the apoptosis receptors on the tumor cell membrane, and it also upregulates the expression and activity of p53 to increase the tumor cell apoptosis. Though possessing diverse pharmacologic effects and safe at high doses, curcumin is limited in application for its poor bioavailability.

##### 2.3.1.5 Nitidine chloride

Nitidine chloride, a quaternary ammonium alkaloid, has been found to exhibit antitumor effects. In the ovarian cancer cell line SKOV3, NC treatment has been reported to inhibit cell growth, cause cell cycle arrest, and induce cell apoptosis. Downregulation of Skp2 mRNA and protein levels has been indicated as the mechanism by which the effect of nitidine chloride manifests (Mou et al., 2017).

##### 2.3.1.6 Safranal

Safranal, a monoterpene aldehyde, is isolated from *Crocus sativus* (saffron) (Table 1). Treatment with safranal has been reported to inhibit the re-activation of quiescent prostate cancer cells. The underlying mechanism of this activity may be that safranal inhibits phosphorylation of AKT and suppresses the transcriptional activity of E2F1 and NF-κB, leading to downregulation of Skp2 expression at both the mRNA and protein levels as well as downregulation of other cell-cycle-related proteins (Table 2) (Jiang et al., 2020).

##### 2.3.1.7 5gg

1,2,3,4,6-penta-O-galloyl-β-D-glucose (5 gg), a natural polyphenolic compound, is abundant in *Oenothera paradoxa* (Table 1). It has been reported that combination treatment with chrysin and 5 gg inhibits breast cancer cell proliferation *in vitro* and *in vivo* by downregulating phospho-LRP6 and Skp2 proteins (Huang et al., 2015). In 5 gg treated MDA-MB-231 cells, a striking decreased in Skp2 mRNA was observed, as well as a significant decrease in Skp2 protein level, indicating that decrease in Skp2 protein partly in transcriptional level (Huang et al., 2011).

##### 2.3.1.8 Lycopene

Lycopene, a bioactive carotenoid, is most abundant in tomatoes. Lycopene has been reported to downregulate Skp2 expression and inhibit cell growth in MDA-MB-231 cells, however, downregulation of Skp2 does not correlate with p27 upregulation upon lycopene treatments (Table 1) (Huang et al., 2011). Like *5* gg, in lycopene treated MDA-MB-231 cells, a significant decreased in Skp2 mRNA and protein level was observed.

##### 2.3.1.9 Diosmetin

Diosmetin, a phytoestrogen, can be extracted from medicinal herbs, citrus fruits, and oregano. Diosmetin treatment has been reported to inhibit Skp2 expression transcriptionally, and it also downregulates Skp2 expression *via* inhibition of the SKP2/Bcr-Abl pathway, exhibiting antitumor activity in CML cells and xenograft models (Liu et al., 2020a) (Table 2).

#### 2.3.2 Post-translational inhibition

##### 2.3.2.1 Dioscin

Dioscin, a steroidal saponin, is an active ingredient in some traditional Chinese medicines. It has been found in Liuwei Dihuang decoction and *Dioscoreae* rhizome extracts (Tao et al., 2018). Dioscin was identified as an inhibitor of glucose consumption and lactate production in CRC screening from 88 natural products (Zhou et al., 2020). Dioscin has been demonstrated to enhance the interaction between Skp2 and Cdh1, promoting Skp2 ubiquitination and degradation, and finally decreasing glucose consumption and lactate production *in vitro,* while treatment with Dioscin has also been shown to inhibit CRC tumor growth *in vivo*.

##### 2.3.2.2 Flavokawain A

The chalcone flavokawain A (FKA) is extracted from the kava plant (Table 1). It has been reported to decrease Skp2 protein level by inhibiting conjugations between NEDD8 and Cullin1. FKA has been reported to inhibit cell proliferation in a dose-dependent manner by decreasing Skp2 protein post-translationally in numerous tumors(Table 2), including pRb-deficient prostate cancer cells, bladder cancer cell lines (Tang et al., 2008), HER2-overexpressing breast cancer cell lines (Jandial et al., 2017), synovial sarcoma cell lines (Wang J. et al., 2020), and osteosarcoma cell lines (Zhang et al., 2018). FKA treatment also inhibits tumor formation and metastasis in autochthonous transgenic adenocarcinoma of the mouse prostate (Li et al., 2015).

##### 2.3.2.3 Gartanin

Gartanin, a 4-prenylated xanthone, is isolated from mangosteen (Table 1). Gartanin was reported to dock onto the regulatory subunit of NEDD8-activating enzyme (NAE) complex and next to the NEDD8 binding complex, leading to inhibit of conjugation of NEDD8 and Cullin1, finally promoting degradation of Skp2 protein in ubiquitination manner. It has been reported to be a cell-active ubiquitination inhibitor in prostate cancer that selectively degrades Skp2 and upregulates FBXW2 expression, thus inducing autophagy and inhibiting cancer cell growth. In 22Rv1 and PC3 cells, treatment with gartanin for 24h downregulates Skp2 protein expression dose-dependently(Pham et al., 2020) (Table 2).

##### 2.3.2.4 Quercetin

The flavonoid quercetin is abundant in vegetables and fruits (Table 1). In quercetin treated MDA-MB-231 cells, cell growth was inhibited by downregulation of Skp2 protein level, however, no significant changes in Skp2 mRNA levels were observed in these cells, indicating that quercetin-induced Skp2 protein level decrease might involve a post-transcriptional mechanism (Huang et al., 2011).

#### 2.3.3 Disruption protein interaction

##### 2.3.3.1 Betulinic acid

Betulinic acid, a plant-derived Skp2 inhibitor, has been demonstrated to exhibit antitumor activity and low cytotoxicity in Phase 1 clinical trials (NCT00701987 and NCT030904511) against cutaneous metastatic melanoma. BA was identified as an Skp2 inhibitor from a library of 841 phytochemicals by high-throughput structure-based virtual screening (Table 1). It has been demonstrated to bind to Skp2 by forming H-bonds with the Lys145 residue, decreasing Skp2 stability, preventing Skp1-Skp2 interaction and thus inhibiting SCF^Skp2^ E3 ligase, leading to decreased tumor proliferation and migration in non-small-cell lung cancer (He et al., 2021). In the Phase 1 clinical trials of NCT00701987), sponsored by Advanced Life Sciences, 12 patients with cutaneous metastatic melanoma were included. However, no outcome or status was updated until recently.

##### 2.3.3.2 Linichlorin A and gentian violet

Linichlorin A, a sesquiterpene lactone, was isolated from *Centaurea linifolia* Vahl (Table 1). Gentian violet (GV), also known as crystal violet or methyl violet, is a triphenylmethane dye. Linichlorin A and Gentian violet was screened out as small (Table 1) inhibitors of p27^Kip1^ degradation by a high-throughput screening system from approximately 20,000 compounds in the RIKEN NPDepo chemical library. Linichlorin A and Gentian violet were selected out as inhibitors of protein-protein interaction between Skp2-Cks1 and p27^Kip1^, and they were demonstrated to inhibited p27^Kip1^ ubiquitination, leading to inhibition of cell growth and delay of cell cycle progression in human cervical cancer cells and murine breast cancer cells (Table 2) (Ooi et al., 2013). Recently, Linichlorin A was also reported to induce growth inhibition in four human cancer cell lines, by the mechanisms associated with cytochrome c release and poly (ADP-ribose) polymerase cleavage (Estevez-Sarmiento et al., 2020). However, GV has always been reported to have multiple functions, including antibacterial, antifungal, anti-helminithic, anti-trypanosomal, anti-angiogenic and antitumor functions (Maley and Arbiser, 2013).

## 3 Discussion

The IC_50_ data of the Skp2 inhibitors or inhibiting compounds in various kinds of tumors at cellular level were collected in Table 2. In prostate cancer cell lines PC3 and LNCaP, the IC_50_ values of Skp2 inhibitor SZL-P1-41 (i.e., #25) was 5.61 μM and 1.22 μM respectively (Chan et al., 2013), much lower than the natural Skp2-inhibiting compounds Gartanin (13.56 μM, 8.32 μM) (Pham et al., 2020) and Safranal (0.512 mM, 0.234 mM)(Jiang et al., 2020), and also lower than SMIP004(40 μM in LNCaP)(Rico-Bautista et al., 2010), a non-structure-based compounds. However, the treatment time course was quite different between these compounds, with 4 days for SZL-P1-41, 48 h for Safranal, and 72 h for Gartanin, which might lead to a bias of the IC_50_ in this comparation.

**FIGURE 1:**
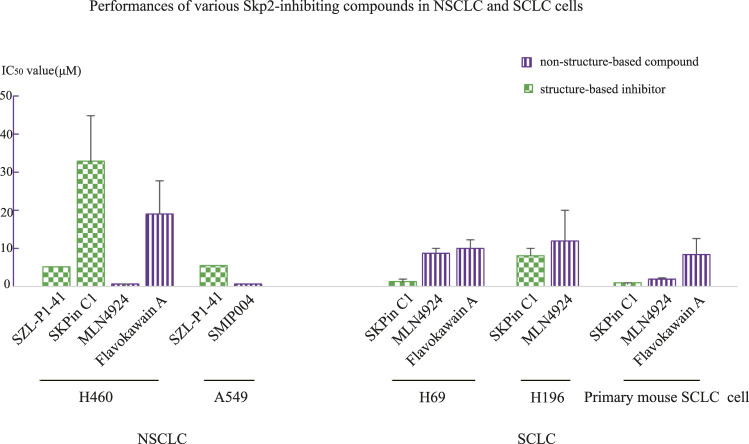
Comparison of IC_50_ values between structure-based Skp2 inhibitors and non-structure-based compounds in NSCLC and SCLC cell lines.

This phenomenon that structure-based inhibitor of Skp2 was more efficient than non-structure-based compounds, was also observed in lung cancer cell line H520, H69, H196 and primary mouse small cell lung cancer (SCLC) cell, in which IC_50_ value of Skp2 inhibitor SKPin C1 was lower than MLN4924 and Flavokawain A (Zhao et al., 2020). H69 and H196 are both SCLC cell lines, so we investigated the data of non-small cell lung cancer (NSCLC) cell lines. In H460 and A549 cell lines, the Skp2 inhibitors did not show strong inhibitor effects. The MLN4924 had the lowest IC_50_ values in H460(0.43 μM), lower than Skp2 inhibitor SZL-P1-41(5.15 μM) and SKPin C1(33 μM). SMIP004 also exerted stronger inhibitor effects than SZL-P1-41, with IC_50_ value of 500 nM at 24 h (Zhao et al., 2020). Flavokawain A and SMIP004 was Skp2-inhibiting compound, while MLN4924 is an SCF^Skp2^ complex neddylation inhibitor which might not bind with Skp2. In the above comparisons, the SZL-P1-41 was also with the longest treatment time course of 4 days, however, it’s not the potent inhibitor with IC_50_ value higher than other inhibitor or compounds, so we ignore the bias of the IC_50_ lead by the differences of treatment time courses, and concentrate on the potent compounds with much lower IC_50_ values and shorter treatment time. The different performances between Skp2 inhibitor and Skp2-inhibiting compounds in SCLC cell lines and NSCLC cells lines (Figure 1), might provide a clue that Skp2 played more important roles in SCLC than in NSCLC cells from the point of tumor pathological mechanisms. Furthermore, when treating lung cancers, different Skp2 targeting compound with respective effects should be selected according to the type, stage, pathological mechanisms of lung cancers. However, the wild-type RB1 existence should also be taken into consideration, for instance, the IC_50_ value of SKPin C1 in lung squamous cell carcinoma cell line H520 with wild-type RB1 was higher than that in SCLC cell line H69 with deficient RB1 (Zhao et al., 2020). As lung cancer cells were quite heterogenous, we should take these data seriously.

In addition, in T cell leukemia cell line TAIL7, two Skp2 inhibitors, SZL-P1-41(30 μM) and SKPin C1(2.4 μM) exhibited significantly different performances (Rodriguez et al., 2020). SZL-P1-41 binds to the Trp97 and Asp98 residues of Skp2, preventing Skp2–Skp1 interaction, while SKPin C1 targets the Skp2-p27 interaction interface to block p27 degradation. These data imply that in IL-7-dependent T-ALL cell line TAIL7, preventing a specific substrate(p27) degradation might exert stronger inhibitor effects than inhibition of Skp2 function by disrupting the SCF^Skp2^ complex. However, this phenomenon was not observed in other tumor cell lines listed in Table 2. The IL-7 signaling pathway was proved to play important roles in Notch/Skp2/p27^Kip1^ pathway to promote Skp2 induction in T-ALL development, and which might attribute to this observation that SKPin C1 exerted more inhibitory effects on TAIL7 than SZL-P1-41. This result indicated that one should choose Skp2 inhibitor compound according to the mechanisms of various cancers, and specific target should be selected according to the pathological mechanisms of specific cancer when developing drugs. Somehow, it might have stronger inhibitor effects when only block the binding between Skp2 and one specific substrate, other than inhibiting the function of the SCF^Skp2^ complex in some situation.

To the contrary, in NSCLC cell line H460, SZL-P1-41(5.15 μM) exerted stronger inhibitory effects than SKPin C1(33 μM) (Zhao et al., 2020), indicating that in lung cancer with same mechanisms of H460, disrupting the SCF^Skp2^ complex prevented tumor development more significantly than only preventing p27 degradation. Other Skp2 substrate proteins might also play important roles in lung cancer other than p27, implying the multiple roles of Skp2 in cancer development.

Natural Skp2 inhibitor should be considered additionally. A natural Skp2 inhibitor with high affinity and specificity, which could disrupt the binding between Skp2 and other proteins, might be a potential effective Skp2 inhibitor in tumor treatment, or used as template for developing more potent and safer drugs. For example, natural Skp2 inhibitor Betulinic acid performed so well to be used in clinical trials (He et al., 2021). According to the IC_50_ data in Table 2, in several cancer cell lines, the Skp2 inhibitor exerted stronger inhibitory effects than the Skp2-inhibiting compounds, especially the natural Skp2 inhibitor gentian violet, which disrupted the binding between Skp2 and p27, with a IC_50_ of 0.4 μM in human cervical cancer cell HeLa, and 0.6 μM in murine breast cancer cell tsFT210, comparing to the 5.3 μM in normal NIH3T3 cells (Ooi et al., 2013). However, the decreased IC_50_ values of many natural inhibitors should be considered carefully. For instance, the gentian violet also has other cellular toxic functions in addition to Skp2 inhibitor, such as the genetic toxicity, uncoupling effect on mitochondrial oxidative phosphorylation, photodynamic action (Moreno et al., 1988). The low IC_50_ value of gentian violet might be attributed to high permeability or non-specific off target toxicity, other than the direct effects on Skp2. There is another situation, many natural drugs always have multiple targets or multiple pharmacologic effects by affecting several signaling pathways. Take curcumin, for example, the observed tumor inhibition effects of curcumin might attribute to several inhibitor mechanisms including inhibition of Skp2. The really inhibitory effect of natural Skp2 inhibitor should be considered carefully. In conclusion, more confirmative tests should be carried out to evaluate the potence of natural inhibitors.

There are also two interesting findings. The first one, when treating primary mouse SCLC lung cell and primary mouse SCLC liver metastatic cell, the IC_50_ value of SKPin C1 was almost the same, however, the IC_50_ value of MLN4924 decreased from 1.8 μM in primary mouse SCLC lung cell to 0.18 μM in primary mouse SCLC liver metastatic cell (Zhao et al., 2020). MLN4924 is an inhibitor of NEDD8-activating enzyme (NAE), which inhibits the function of SCF^Skp2^ complex by inactivation of cullin-RING ligases (Soucy et al., 2009). However, it also has some neddylation-independent activities, such as activating EGFR, promoting glycolysis, inhibiting IFN-β production, and activating the JNK signaling pathway (Jin et al., 2013). The different performances of MLN4924 in primary mouse SCLC lung cell and primary mouse SCLC liver metastatic cell, implied that some important mechanisms might change during SCLC cell metastasis, and this mechanism changes might relate to neddylation-dependent activities of MLN4924, indicating that SCF^Skp2^ or other E3 neddylation might promote the SCLC cell metastasis, other than only Skp2 protein or the ubiquitination mediate by Skp2. In addition, this mechanism changes might also relate to neddylation-independent activities of MLN4924, indicating other signaling pathways might play important roles in SCLC cell metastasis process, which could be used as target for drug development or therapy potentially.

Another interesting finding is that when treating LNCaP cells with SMIP004, the IC_50_ value decreased from 40 μM in LNCaP cells with normal Skp2 expression level (LNCaP-WT) to 1.09 μM in LNCaP cells with stable Skp2 overexpression (LNCaP-S14) (Rico-Bautista et al., 2010). In this study that SMIP004 was selected out, the increased Skp2 protein level was proved to be associated with the inhibitory effect of SMIP004. Recently, SMIP004 was reported to mediate a mitochondrial pathway (Rico-Bautista et al., 2013), leading to mitochondrial ROS formation, unfolded protein response activation and apoptosis finally. These results implied that Skp2 might also relate to the apoptosis induced by ROS and unfolded protein response, supplying a new clue for Skp2-targeting therapy. The previous reported findings were consistent with the roles of Skp2 in ROS generation and unfolded protein response. Skp2 has been reported to relate to the degradation of Myc which is necessary for the generation of ROS mediated by pre-existing Romo1 protein (Lee et al., 2011). The decreased Skp2 protein level was reported to relate with the unfolded protein response as well (Han et al., 2013).

**FIGURE 2:**
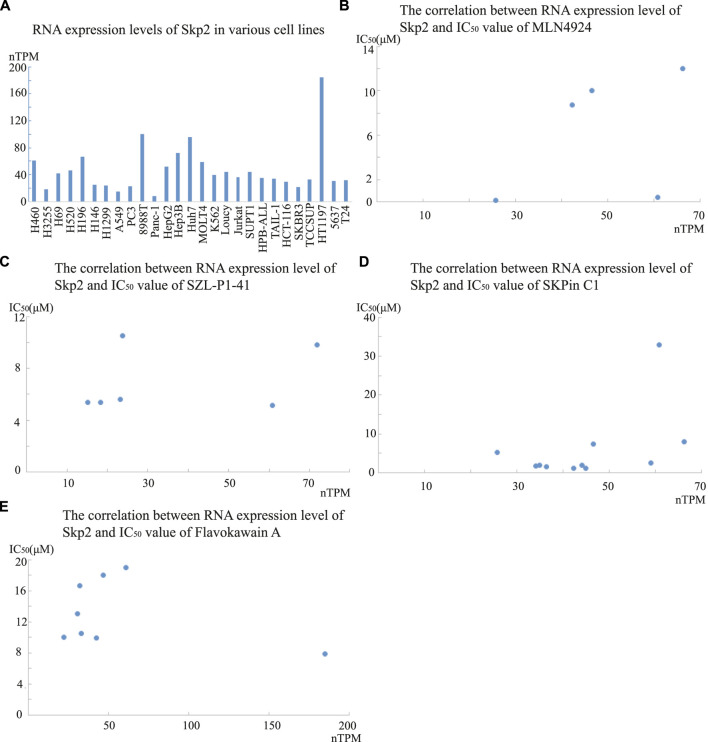
The RNA expression levels of Skp2 in various cell lines, and its correlation with IC_50_ values of four Skp2 inhibiting compounds. A The RNA expression level of Skp2 in various cell lines in Human Protein Atlas database; B-E. The correlation between RNA expression level of Skp2 and IC_50_ values of SZL-P1-41, MLN4924, Skpin C1, and Flavokawain A respectively. The RNA expression level data was collected from Human Protein Atlas database, and the IC_50_ value data was collected from the references listed in Table 2.

Furthermore, the RNA expression of Skp2 showed low cancer specificity, and we collected the data of Skp2 RNA expression level in several of the cell lines in Table2 from the Human Protein Atlas database, with the data represented as nTPM (Figure 2A). The correlation between the RNA expression level and IC_50_ value was analyzed in treatment groups of SZL-P1-41, MLN4924, Skpin C1, and Flavokawain A. No apparent significant correlation was observed (Figure 2B-E). However, the mRNA level might not always consistent with the Skp2 protein level, the correlation between protein level and IC_50_ value should also be analyzed in the future when protein expression data is accessible. Meanwhile, one of the Skp2 key substrate protein, p27, was reported to relate with clinical stages and chemoresistance of various tumors. In hepatocellular carcinoma, the p27 protein level was significantly lower in advanced staged of disease (Tannapfel et al., 2000). In various other cancers, the protein level of p27 was also reported to be decreased in advanced tumor stage and relate to poor prognosis. In prostate cancer, for instance, the intensity of p27 expression was negatively associated with the tumor stage and Gleason scores, and the upregulation of p27 expression was observed in prostate adenocarcinoma cells after hormonotherapy (Nikoleishvili et al., 2007). In addition, p27 protein level was lower in breast cancer cell line MDA-MB-231 compared to BT474, and the MDA-MB-231 cells showed higher susceptibility to treatment of 5 gg, curcumin, and lycopene respectively, comparing to BT474. However, BT474 was more sensitive to quercetin (Huang et al., 2011). This observation implied that different mechanisms involve in the tumor development as MDA-MB-231 is ER/HER2-negative and BT474 was HER2-positive. Furthermore, in SCLC tissues, the observation that protein level of p27 was suppressed and protein level of Skp2 was increased was demonstrated by Immunohistochemistry assay (Zou et al., 2022). In NSCLC tissues, the total positive rate of p27 protein was significantly lower than in non-cancerous lung disease, meanwhile, the positive rate of p-AKT was significantly higher than these non-cancerous controls. Protein p-AKT was another important substrate of Skp2, which mediates the K63-linked ubiquitination of p-AKT rendering AKT more stable (Miao et al., 2006). The positive expression rate of AKT was also significantly higher in other cancer tissues, such as gastric cancer tissues compared to normal gastric mucosa (Gu et al., 2014). Skp2 has been reported to be the ubiquitin ligase not only for p27 but also other antioncogenic proteins, and it has other important oncogenic effects, suggesting the reduction of Skp2 level is a rational therapeutic method.

As a hot research spot for therapeutic target-drugs of cancers, inhibition of Skp2 by genetic deletion or pharmacological blockade showed little effect on normal tissues based on murine models, indicating that Skp2 inhibition may not induce serious adverse effect (Chan et al., 2010; Lin et al., 2010). Recently, Skp2 has been reported to be a therapeutic target in other non-cancer diseases, such as BM transplantation (Wang et al., 2011), depression (Wang D. et al., 2019). However, we should still consider the harmful aspect of Skp2 inhibition treatment seriously. For example, the Skp2 inhibition might affect the female patient’s fertile ability. The recurrent miscarriage patients were reported to have reduced expression in the decidual tissues compared to normal pregnant women, and Skp2-deficient mice showed impaired ovarian development and reduced fertility (Lv et al., 2021). The slowdown of cell cycle relates to cellular senescence closely, and the Skp2 inhibition induces the senescence of endothelial progenitor cell (EPC) (Wang H. H. et al., 2020), and inhibits angiogenic senescence, leading to aging-related vasculopathy, which finally affects atherosclerosis related disease or vascular event.

Until recently, there are emerging various Skp2-inhibiting compounds with different inhibitory mechanisms. Comprehensively considering the characteristics of Skp2 protein, such as the self-amplifying feedback loop of itself and potentially side effects of Skp2 inhibition (Yung et al., 2007), it could be suggested to adopt two or more Skp2-inhibiting compounds for Skp2 inhibitory treatment clinically, which would be a better way in tumor treatment than single Skp2 inhibitors. The choice of Skp2 inhibitor or inhibiting compound should be based on the disease pathological mechanisms. For example, the expression and stability of Skp2 protein was reported to relate to the vemurafenib-resistance in melanoma cells. Vemurafenib inhibits Skp2 on transcriptional level, and b-AP15 inhibits Skp2 on post-translational level. Combined use of both inhibitors is effective in treating vemurafenib-resistant melanoma (Wu et al., 2022). In addition, the combination of tubulin inhibitor VERU-111 and vemurafenib also inhibited vemurafenib-resistant melanoma effectively by inhibiting tubulin protein and Skp2 protein together (Cui et al., 2021). Another example, the SCF^Skp2^ components related to BTZ resistance, and combined use of BTZ and Skp2 inhibitor inhibits BTZ-resistant multiple myeloma growth, rendering targeting SCF^Skp2^as a strategy to overcome therapeutic resistance in multiple myeloma.

## 4 Future perspectives

Skp2 is overexpressed in various human cancers, and as a component of SCF^Skp2^ E3 ligase, it has been defined as an oncogene. Exploring the functions and pathological mechanisms of Skp2 in tumorigenesis and development could help us achieve a better understanding of tumors and provide new strategies for tumor treatment. The development of drugs that target Skp2 is a promising strategy for future cancer therapy.

In this review, we have summarized recently reported synthetic and natural Skp2 inhibitory compounds. These compounds inhibit Skp2 function or downregulate its expression by various mechanisms, and their effects have been evaluated *in vivo* by animal experiments. However, only the nature Skp2 inhibitor, Betulinic acid, was used in Phase 1 clinical trials to treat cutaneous metastatic melanoma in 2008. Two TKIs, Imatinib and GNF-5, which have been proved for clinical treatment, have been reported to suppress the expression of Skp2 indirectly *via* inhibiting its upstream factor. In addition, the BRAF^V600E^inhibitor vemurafenib has been found to decrease Skp2 mRNA expression and has been used in several clinical trials for the treatment of cancers with BRAF mutation. These TKIs and vemurafenib was not the inhibitor of Skp2, however, these findings implied that targeting upstream factors of Skp2 might be a clue for Skp2 suppression in drug development.

In addition, not all the roles of Skp2 in cancer are fully understood. Additional functional and mechanistic studies of Skp2 are required, and more small molecules that target Skp2 are also needed, especially those that disrupt Skp2 stabilization and function without cytotoxic effects. However, due to the multiple functions of Skp2 in cancers, the cancer type, stage and pathological mechanisms should be taken into consideration when selecting Skp2-inhibiting compound for cancer treatment.
